# Anti-Rheumatoid Arthritic Effects of Paris Saponin VII in Human Rheumatoid Arthritis Fibroblast-Like Synoviocytes and Adjuvant-Induced Arthritis in Rats

**DOI:** 10.3389/fphar.2021.683698

**Published:** 2021-05-28

**Authors:** Mei Meng, Zhenggang Yue, Lu Chang, Yanru Liu, Jinhang Hu, Zhongxing Song, Zhishu Tang, Rui Zhou, Changli Wang

**Affiliations:** ^1^ State Key Laboratory of Research and Development of Characteristic Qin Medicine Resources (Cultivation), Co-Construction Collaborative Innovation Center for Chinese Medicine Resources Industrialization by Shaanxi and Education Ministry, Shaanxi University of Chinese Medicine, Xianyang, China; ^2^ Country School of Pharmacy, Shaanxi University of Chinese Medicine, Xianyang, China

**Keywords:** paris saponin VII, rheumatoid arthritis fibroblast-like synoviocytes, apoptosis, mitochondrial pathway, MAPK pathway, adjuvant-induced arthritis

## Abstract

In the pathogenesis of rheumatoid arthritis (RA), rheumatoid arthritis fibroblast-like synoviocytes (RA-FLS) have tumor-like characteristics, mainly manifested by hyperproliferation and resistance to apoptosis and then it will erode the bone and cartilage, eventually leading to joint destruction. Paris saponin VII (PS VII) is an active compound derived from a traditional herbal medicine named *Trillium tschonoskii* Maxim, which has anti-tumor, analgesic, and immunomodulatory effects. However, its anti-RA effect has not yet been reported. This study was to investigate the effect of PS VII on two rheumatoid arthritis fibroblast-like synoviocytes lines (RA-FLS and MH7A) and adjuvant-induced arthritis (AIA) in rats. *In vitro*, the effects of PS VII on the proliferation, cell cycle, and apoptosis of RA-FLS and MH7A cells were detected by MTT, flow cytometry, and western blot analysis. *In vivo*, the effect of PS VII on the weight of the rat, paw swelling, ankle joint diameter, arthritis index, serum inflammatory cytokines (TNF-α, IL-6, and IL-1β), histopathological assessment and apoptosis proteins in the synovial tissues were evaluated in AIA rats. The *in vitro* studies showed that PS VII inhibited the proliferation of RA-FLS and MH7A cells, induced S phase arrest and triggered cell apoptosis mainly through the mitochondrial apoptotic pathway and the regulation of JNK and p38 MAPK pathways. The *in vivo* studies revealed that PS VII could improve ameliorate body weight, paw swelling, ankle joint diameter, reduce the spleen and thymus index, suppress the production of TNF-α, IL-6 and IL-1β, improve histopathological changes and regulate the expressions of apoptosis proteins in AIA Rats. In conclusion, PS VII could inhibit the proliferation and trigger apoptosis of RA-FLS and MH7A cells by regulating the mitochondrial apoptosis pathway and the JNK and p38 MAPK pathways, and alleviate the symptoms of RA, signifying it to be one of the potential anti-RA therapeutics.

## Introduction

Rheumatoid arthritis is a wide spread autoimmune disease with a high incidence and causes significant disability to those it affects (Petrovská et al., 2021). It is commonly recognized that the pathogenesis of RA involves the infiltration of interstitial inflammatory cells, the tumor-like proliferation of synovial cells, pannus formation, the erosion of bones and articular cartilage (Josef et al., 2018). Of note, synovial hyperplasia is one of the main pathological features of RA, which can lead to the erosion of marginal bone and the destruction of joints (Wei et al., 2020). Moreover, numerous studies have shown that the RA-FLS from RA patients with the characteristics similar to tumor cells, undergoing the tumor-like proliferation, prolonging the cell growth cycle and resisting apoptotic ability, were the main incentives of synovial hyperplasia (Nygaard and Firestein, 2020). According to reports, the RA-FLS from patients had resistance to apoptosis due to the imbalance of anti-apoptotic and pro-apoptotic molecules. Anti-apoptotic mediators such as Bcl-2 and Mcl-2 were up-regulated, while pro-apoptotic proteins such as p53 up-regulated modulator of apoptosis and Bax were down-regulated in RA-FLS (Zhang et al., 2019). The current clinical treatment strategies use non-steroidal anti-inflammatorydrugs (NSAIDs), disease-modifying anti-rheumatic drugs (DMARD), glucocorticoids, and biological agents to improve clinical symptoms, but accompanied by related adverse reactions, such as gastrointestinal bleeding risk, liver and kidney toxicity, infection, and tumor risk (Wang et al., 2018; Conigliaro et al., 2019). Therefore, it is urgent to develop new drugs to inhibit the proliferation and induce apoptosis of RA-FLS, which is one of the critical strategies for the treatment of RA.

In recent years, due to the effectiveness and safety, researchers have paid more attention to natural products (Zhao et al., 2021). Numerous natural products exhibited the anti-RA effect by reducing inflammation, inducing apoptosis, suppressing proliferation, and angiogenesis through modulating MAPK, NF-κB, or mitochondrial-mediated pathways (Zhai et al., 2017; Zhai et al., 2018a; Zhai et al., 2018b; Zhang et al., 2018; Zhai et al., 2019). Currently, several active compounds derived from herb medicines have been used for RA treatment, including Tripterygium Glycosides, Triptolide, and total glucosides of paeony. It has been demonstrated that these compounds could inhibit proliferation and induce apoptosis in RA-FLS, and thus have therapeutic effects on synovial hyperplasia of RA (Jia et al., 2014; Lin et al., 2021a; Lin et al., 2021b). *Trillium tschonoskii* Maxim is a perennial herb of the Trilliaceae, distributed in mid-western China (Li et al., 2005). PS VII (CAS number: 68124-04-9) is one of the main active ingredients extracted from *Trillium tschonoskii* Maxim, which exerted marked cytotoxic effects on several types of cancer cells (Li et al., 2014a; Li et al., 2014b; Zhou et al., 2019; Lin et al., 2021b). The mechanism studies showed that PS VII could induce apoptosis and cell cycle arrest, through regulating the Ras signaling pathway, thus inhibiting the growth of colorectal cancer cells (Li et al., 2014b). As described above that the similar characteristics between RA-FLS and tumor cells, we deduce PS VII may have potential anti-RA effects. The AIA model is a reliable and reproducible experimental model of polyarthritis. Because of its similar pathology to RA, it has been widely used in preclinical evaluation of anti-RA drugs (Yang et al., 2020). In the present study, we investigated the therapeutic effect of PS VII on rheumatoid arthritis fibroblast-like synoviocytes and AIA rats.

## Materials and Methods

### Reagents

MTT, methotrexate (MTX) and Complete Freund’s adjuvant (CFA) was purchased from Sigma-Aldrich (St. Louis, MO, United States). DMEM, phosphate buffer saline (PBS), penicillin, streptomycin, and trypsin were purchased from HyClone Laboratories Inc., (Logan, UT, United States). Fetal bovine serum (FBS) was purchased from Thermo Fisher Scientific, Inc., (Waltham, MA, United States). Dimethyl sulfoxide (DMSO) and Annexin V-FITC Apoptosis Assay Kit were purchased from Absin Bioscience Inc., (Shanghai, China). Hoechst33342/PI cell apoptosis staining kit was purchased from Beijing Solarbio Science and Technology Co., Ltd., (Beijing, China), Cell Cycle Detection Kit, RIPA lysis buffer, the p38 inhibitor (SB203580), and the JNK inhibitor (SP600125), were purchased from Beyotime Biotechnology (Shanghai, China). BCA Protein Quantification Kit was purchased from Tiangen Biochemical Technology Co., Ltd., (Beijing, China). Enhanced chemiluminescence (ECL) developer was purchased from Boster Biological Technology Co., Ltd., (Wuhan, China). Tripterygiumglycosides (TG) were purchased from Yuanda Pharmaceutical Huangshifei Pharmaceutical Co., Ltd., (Huangshi, China). TNF-α, IL-6, and IL-1β ELISA kits were purchased from Neobioscience Technology Co., Ltd., (Shenzhen, China).

PS VII was isolated from the roots and rhizomes of *Trillium tschonoskii Maxim*, and finally separated by HPLC with a purity of >99% (Qinba Moutains, Shaanxi, China) in our laboratory (Li et al., 2013). PS VII was dissolved in DMSO solution at a concentration of 1 M and diluted with PBS to the required concentration for the experiment.

### Cell Culture and Drug Treatment

RA-FLS cell lines (RA-FLS and MH7A) were purchased from Guangzhou Zhou Jennio Biotech Co., Ltd., (Guangzhou, China). RA-FLS and MH7A were cultured in DMEM containing 10% FBS, 1% penicillin, and 1% streptomycin in a 37°C, 5% CO_2_ incubator with different concentrations of PS VII (0, 0.5, 1, 2.5, 5 μM) or MTX (1 μM) for 24, 48, and 72 h. To determine whether the combination of PS VII and appropriate inhibitors could reverse cell proliferation activity, the cells were first pretreated with SB203580 (p38 inhibitor, 10 μM) or SP600125 (JNK inhibitor, 10 μM). After 48 h, the cells were treated with PS VII (2.5 μM). In subsequent experiments, the effect of PS VII combined with p38 inhibitors or JNK inhibitors on proliferation and apoptosis was evaluated to clarify whether PS VII inhibited cell proliferation and induced apoptosis through the MAPK signaling pathway.

### Cell Viability Assay

RA-FLS and MH7A cells were collected during the logarithmic growth phase and were seeded, with trypsinization, into a 96-well plate at a density of 10 × 10^4^ cells/well and 5 × 10^4^ cells/well, respectively. The cells were incubated in DMEM medium at 37°C, 5% CO_2_ for 24, 48, 72 h. Then, RA-FLS and MH7A cells were treated with the above mentioned drugs. MTT solution was added to the 96-well plate (20 μL/well) and incubated for another 4 h. Add 150 μL DMSO to each well and measured the absorbance at 490 nm with a microplate reader (Thermo Multiskan Sky, Waltham, MA, United States).

### Cell Cycle Assay

Flow cytometry and a cell cycle analysis kit were used to investigate the relative distribution of different stages of the cell cycle. Cells were treated with different concentrations of PS VII for 48 h. Wash with PBS and fix with absolute ethanol at −20°C. The cells were collected, then added 100 μL RNaseA to resuspend the cells. 400 μL of PI was added and let it acted in the dark for 30 min. The content of PI-labeled DNA in the cells was quantified by flow cytometry (BD Biosciences, NJ, United States).

### Hoechst33342/PI Cell Apoptosis Staining Assay

Hoechst33342/PI cell apoptosis staining kit was used to evaluate the specific morphological changes that induced apoptosis in the nucleus. RA-FLS and MH7A cells were seeded into 6-well plates at a density of 10 × 10^4^ cells/mL and 5 × 10^4^ cells/mL, respectively. Cells were treated with different concentrations of PS VII for 48 h. The cells were washed with PBS. Then, 1 ml staining buffer was added to each well. After that, 5 μL Hoechst33342 and 5 μL PI was added and cells were stained in the dark for 20 min at 4°C. Finally, cells were analyzed with an inverted fluorescence microscope (Olympus, IX73) and representative digital images were acquired for analysis.

### Apoptosis Assay

In short, RA-FLS and MH7A cells were treated with different concentrations of PS VII for 48 h, 72 h, and the cells were collected. The cells were washed with pre-cooled PBS, and then added 300 μL 1 × Binding Buffer to resuspend the cells. 5 μL Annexin V-FITC and 5 μL PI was added to stain the cells in the dark for 15 min. Then apoptotic cells were detected using flow cytometry and FlowJo 7.6 software was used to determine the proportions (%) of each subpopulation of cells.

### Western Blot Analysis

Western blot analysis was used to determine changes in protein expression that were associated with PS VII treatment. After treatment with PS VII, the cells were collected and lyzed in RIPA lysis buffer containing protease and phosphatase inhibitors in an ice bath. Afterwards, the supernatant was collected by centrifugation. The total protein concentration in each sample was determined using a BCA protein analysis kit. The protein samples were loaded separated by SDS-PAGE and electrophoretically transferred onto PVDF membranes. Incubate the membrane with blocking solution (5% skimmed milk) at room temperature for 1 h. After washing, incubated with the corresponding primary antibody overnight at 4°C.The primary antibody was used at the following dilutions: Cyclin A (1:1,000), CDK2 (1:1,000), p21 (1:800), p38 (1:1,000), p-p38 (1:1,000), JNK (1:1,000), *p*-JNK (1:1,000), ERK (1:1,000), *p*-ERK (1:1,000), Bcl-2 (1:2000), Bcl-xL (1:1,000), Mcl-1 (1:1,000), Cytochrome C (1: 1,000), Bax (1:4,000), Bad (1:1,500), Cleaved Caspase-9 (1:900), Cleaved Caspase-3 (1:1,000), β-actin (1:3,000), all antibodies werepurchased from Cell Signaling Technology, Inc. (Boston, MA, United States). Incubated with a secondary antibody (Cell Signaling Technology, Inc., Boston, MA, United States) coupled with horseradish peroxidase. Positive antibody binding was then visualized by ECL detection and analyzed by Image J software (BioRad, Hercules, CA, United States).

### Animals

SPF male SD (Sprague-Dawley) rats weighing 140–180 g were purchased from Chengdu Dashuo Experimental Animal Co., Ltd.. All SD rats were raised in a constant temperature (23 ± 2°C) and constant humidity (50 ± 5%) environment, and adequate feed and drinking water were provided. All SD rats were adaptively fed for one week before the experiment. All experimental operations on rats were complied with the regulations promulgated by the International and Local Laboratory Animal Eating and Protection Committee.

### Adjuvant-Induced Arthritis and Drug Administration

Rats were injected subcutaneously with 100 μL of complete Freund’s adjuvant (CFA) into the right hind foot plantar to establish an AIA model. The control group was injected with the same dose of saline. The AIA rats were randomly divided into a model group (Model), a tripterygium glycosides group (TG), and three different doses of PS VII groups. On the 16–40 days after AIA induction, the rats were given intragastrically at the same time point every day. The PS VII group was given different doses of PS VII (2.5 mg/kg, 5 mg/kg, 10 mg/kg) by intragastric administration. The control group and the model group were treated with the same dose of 0.5% CMCeNa solution. According to previous research (Bao et al., 2019), the dose of TG was determined and preliminary experiments were conducted. The TG treatment group was given TG (7.6 mg/kg) every day as a positive drug.

### Determination of Weight, Paw Swelling and Ankle Joint Diameter

During the drug treatment, the body weight and growth status of the rats were measured every 4 days. The YLS-7C paw swelling meter (Jinan Yiyan Technology Development Co., Ltd., Jinan, China) was used to evaluate the toe swelling based on the volume of the right hind paw. Paw swelling (%)=(Vt−Vn)/Vn × 100%, where Vn and Vt are the volume of the right hind paw before and after induction, respectively. In addition, the diameter of the ankle joint of the right hind paw of the rat was measured with a vernier caliper. Before establishing the AIA model, the original weight of the rat, the volume of the right hind paw, and the diameter of the ankle joint must be determined.

### Evaluation of Arthritis Index and Visceral Organ Index of Rats

The pathological changes of arthritis were observed every 4 days from the 12th day. The arthritis index was used to assess the severity of arthritis. 0 points = no significant changes in rat joints; one point = small amounts of erythema in rat joints; two points = slight redness or severe erythema in rat joints; three points = obvious redness and swelling of rat ankle joints; four points = all Severe swelling of the joints. The arthritis index of each rat was the sum of the limbs, and the maximum arthritis score were 16 points. The thymus and spleen of each group of rats were taken, weighed and recorded, and the thymus and spleen index was calculated. Spleen/thymus index = thymus or spleen (g)/weight (g) * 100%

### Determination of the Levels of Inflammatory Cytokines TNF-α, IL-6, and IL-1β in Serum

1 h after the last administration, blood was collected from the abdominal aorta, placed in a vacuum blood collection tube, and allowed to stand at room temperature for 1 h. Serum was separated by centrifugation at 1,238 *g* for 15 min, and stored in a refrigerator at −80°C for later use. The serum supernatant factors TNF-α, IL-6 and IL-1β levels were determined by an enzyme-linked immunosorbent assay (ELISA) kit according to the manufacturer’s instructions.

### Histopathological Examination

The ankle joint was fixed in 4% paraformaldehyde, decalcified in 10% EDTA for 2 months, embedded in paraffin, and cut into thin slices. The sections were stained with hematoxylin and eosin (H and E). Use DM-2500 optical microscope (Germany LEICA, Inc., Wetzlar, Hesse-Darmstadt, Germany) to observe histopathological changes and severity. As mentioned earlier Sims et al.(2004), inflammation, synovial hyperplasia, cartilage damage, and bone erosion are all scored on a scale of 0–4.

### Determination of Proteins in Synovial Tissues

The synovial tissues from each group were obtained as the similar method in the previous work (Bao et al., 2019). Briefly, after disinfected the knee joints, the skin were cutted, the exposed synovial tissue were gently removed. Then, the synovial tissues were rinsed with PBS and stored in liquid nitrogen. To extract the proteins from tissues, a mixture lysis buffer with protease and phosphatase inhibitor were added into the tissue and then homogenized every 5 min for 1 h. Then, the homogenates were centrifugated at 12,000 g for 15 min at 4°C. The total proteins concentrations in the supernatants were detected by BCA protein assay kit. The protein samples were detected by western blot analysis.

### Statistical Analysis

At least three independent repeated experiments, all data were expressed as mean ± SD. All data were analyzed using SPSS 22.0 (SPSS, Chicago, IL, United States) and GraphPad Prism 8.0 (GraphPad Software, Inc., San Diego, CA). One way ANOVA was used for statistical analysis, followed by Bonferroni’s post-test. When *p* < 0.05 between the groups was considered statistically significant.3 Results.

## Results

### PS VII Inhibited the Proliferation of RA-FLS and MH7A Cells

**FIGURE 1:**
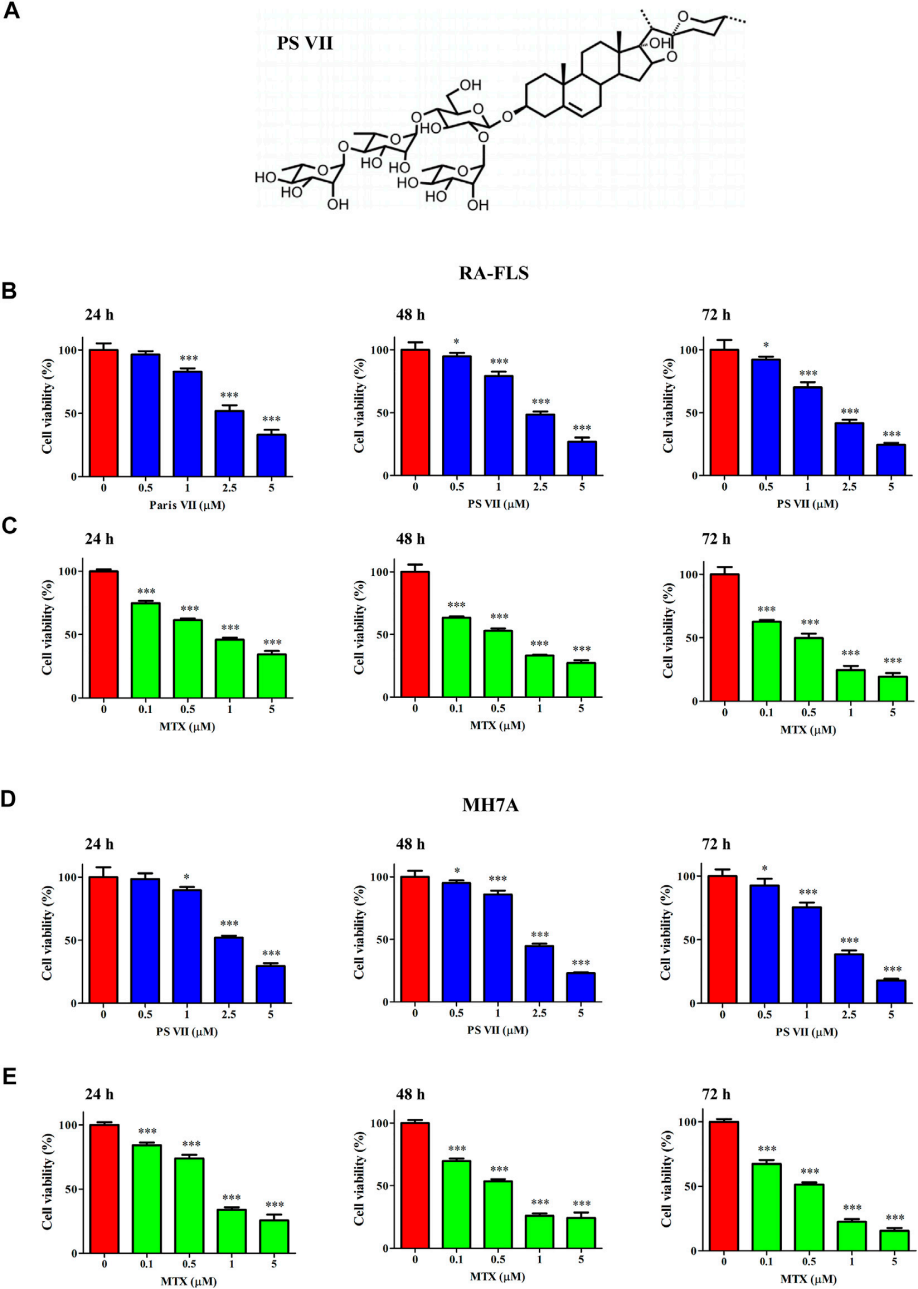
Effects of PS VII on the cell viability of RA-FLS and MH7A cells. **(A)** The molecular structure of PS VII. **(B, C)** The cell viability of RA-FLS was determined by MMT assay after treated with different concentrations of PS VII (0, 0.5, 1, 2.5, 5 μM) and MTX (0.1, 0.5, 1, and 5 μM) for 24, 48, 72 h. **(D, E)** The cell viability of MH7A was determined by MTT assays after treated with different concentrations of PS VII (0, 0.5, 1, 2.5, 5 μM) and MTX (0.1, 0.5, 1, 5 μM) for 24, 48, 72 h. MTX was treated as positive control. Data were expressed as mean ± SD from three independent experiments. *p < 0.05, **p < 0.01, ***p < 0.001.

To evaluate the cytotoxicity of PS VII in RA-FLS and MH7A cells, the cell viability was measured after treatment with multiple concentrations of PS VII for 24, 48, 72 h (Figure 1). MTT analysis showed that the 0.5 μM PS VII group had no significant difference in cell viability, compared with the control group. PS VII with concentrations higher than 0.5 μM had the great cytotoxic effect in the cells at 24 h (Figures 1B,D). The treatment of RA-FLS and MH7A cells with 2.5 μM PS VII for 24 h resulted in a 48.12 and 48.03% loss of cell viability, respectively. Moreover, there were also obvious differences in the cell viability under 0.5 μM PS VII at 48 h or 72 h compared with the control group. MTX, which could inhibit the proliferation of RA-FLS and MH7A cells, was used as a positive control (Figures 1C,E). These results demonstrated that PS VII and MTX treatment reduced cell proliferation in a dose- and time-dependent manner.

### PS VII Induced Cell Cycle Arrest in S Phase and Modulated Corresponding Cyclins in RA-FLS and MH7A Cells

**FIGURE 2:**
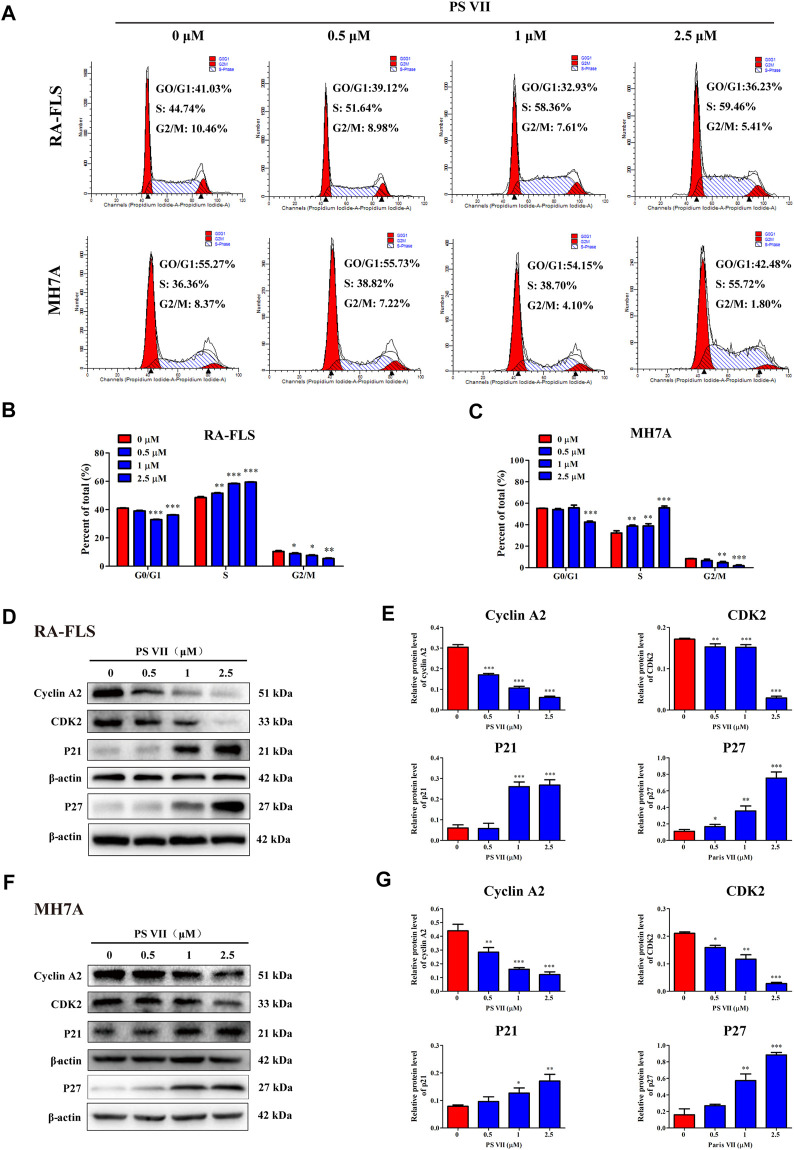
Effects of PS VII on cell cycle in RA-FLS and MH7A cells. RA-FLS and MH7A cells were treated with PS VII (0, 0.5, 1, 2.5 μM) for 48 h. **(A)** Cell cycle distribution was assessed by flow cytometry. **(B, C)** The proportion of cells in G0/G1, G2/M, and S phases. **(D, F)** Expression levels of the S phase related proteins cyclin A2, CDK2, P21, P27 were detected by Western blot. **(E, G)** The relative expression of protein in RA-FLS and MH7A cells was determined by optical density analysis. Image J was used to determine the density ratio of each protein band relative to β-actin. Compared with the control group, *p < 0.05, **p < 0.01, ***p < 0.001.

Cell proliferation is regulated by cell cycle progression. The cell cycle was a process of cell division and replication, consisting of G0, G1, S, and G2/M phases (Strzyz, 2016). To investigate whether the inhibitory effect of PS VII on cell proliferation was caused by cell cycle arrest, the effect of PS VII on nuclear DNA content was detected by flow cytometry. As shown in Figures 2A–C significant proportion of RA-FLS and MH7A cells treated with PS VII (0.5, 1, 2.5 μM) were held in S phase. Compared with the control group, the proportion of S phase cells in RA-FLS and MH7A cells increased from 44.74 to 59.46% and from 36.36 to 55.72%, respectively. The proportion of cells in the G0/G1 and G2/M phases decreased. This tendency was more evident in the high-dose group (2.5 μM PS VII), indicating that PS VII treatment induced S phase arrest in both cells. The proteins cyclin A2, CDK2, P21, and P27 are crucial molecules for S phase cell cycle arrest. As shown in Figures 2D–G, PS VII decreased the expression of cyclin A2, CDK2 protein and increased the protein expression of P21 and P27 in RA-FLS and MH7A. These results demonstrated that the PS VII induced S phase arrest was involved in modulating the expression of cyclin A2, CDK2, P21, and P21.

### PS VII Induced Morphological Changes in RA-FLS and MH7A Cells

**FIGURE 3:**
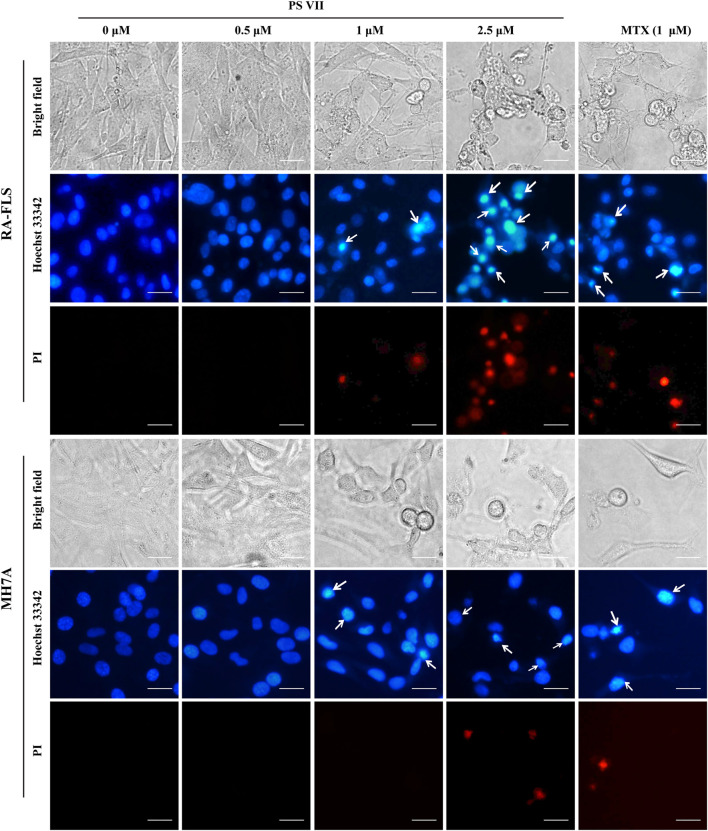
Effects of PS VII on morphological changes in RA-FLS and MH7A cells. Cells were treated with PS VII for 48 h. Nuclear morphology was stained with Hoechst33342/PI cell apoptosis staining assay and was observed under inverted fluorescence microscope, the white arrow indicates the chromatin concentration and nuclear fragmentation in the cells, microscope magnification × 400, scal bar = 20 μM.

Hoechst33342/PI cell apoptosis staining assay was used to evaluate the specific morphological changes that induced apoptosis in the nucleus. As shown in Figure 3, the normal RA-FLS and MH7A cells were long spindle shape, and the nuclei morphology were blue uniform. Chromatin accumulation and nuclear condensation occurred in both RA-FLS and MH7A cells after PS VII treatment for 48 h. The results indicated that PS VII treatment induced apoptosis in RA-FLS and MH7A.

### PS VII Triggered Cell Apoptosis in RA-FLS and MH7A Cells

**FIGURE 4:**
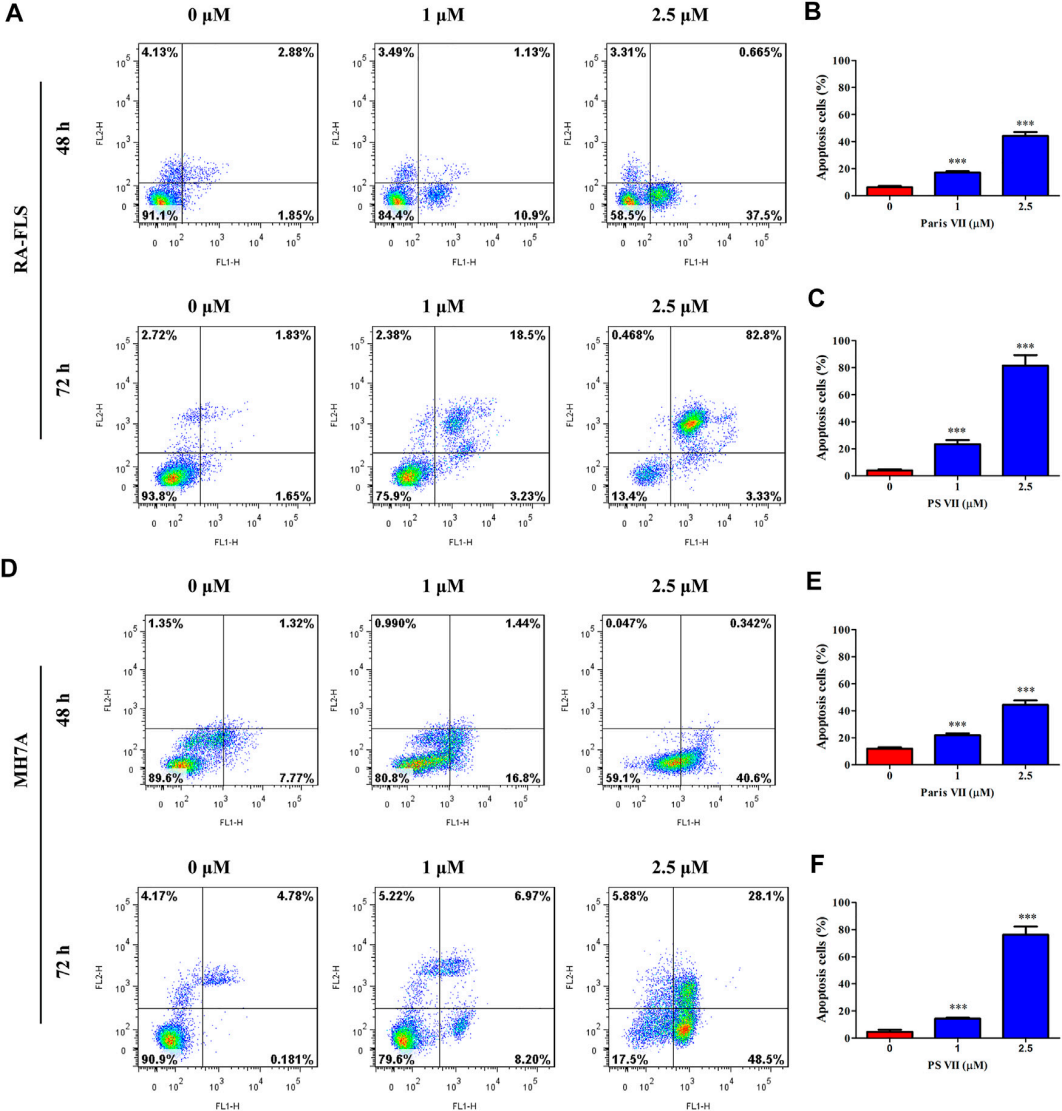
Effects of PS VII on apoptosis in RA-FLS and MH7A cells. RA-FLS and MH7A was treated withdifferent concentrations of PS VII (0, 1, 2.5 μM) for 48 h, and 72 h. The apoptosis was determined by flow cytometry. **(A)** The apoptosis of RA-FLS with PS VII treatment for 48 h (A, upper panel) and 72 h (A, lower panel). The populations of apoptotic cells were evaluated as the percentage of total cells for 48 h **(B)**, and 72 h **(C)**. **(D)** The apoptosis of RA-FLS with PS VII treatment for 48 h (A, upper panel) and 72 h (A, lower panel). The populations of apoptotic cells were evaluated as the percentage of total cells for 48 h **(E)**, and 72 h **(F)**. Each bar represents the mean ± SD of three independent experiments. Compared with the control group, *p < 0.05, **p < 0.01, ***p < 0.001.

To further illustrate whether PS VII treatment could induce cell apoptosis in RA-FLS and MH7A cells, the Annexin V-FITC/PI apoptosis detection kit was used, and the apoptosis was detected by flow cytometry. As shown in Figure 4A–F, PS VII treatment mainly induced the early apoptosis in RA-FLS and MH7A for 48 h, and the late apoptosis was obviously occurred after 72 h. A comparison of the total ratio of apoptosis cells (early and late apoptotic cells) confirmed that PS VII triggered apoptosis in RA-FLS and MH7A.

### PS VII Affected the Expression of Proteins Associated With the Mitochondrial Apoptosis Pathway in RA-FLS and MH7A Cells

**FIGURE 5:**
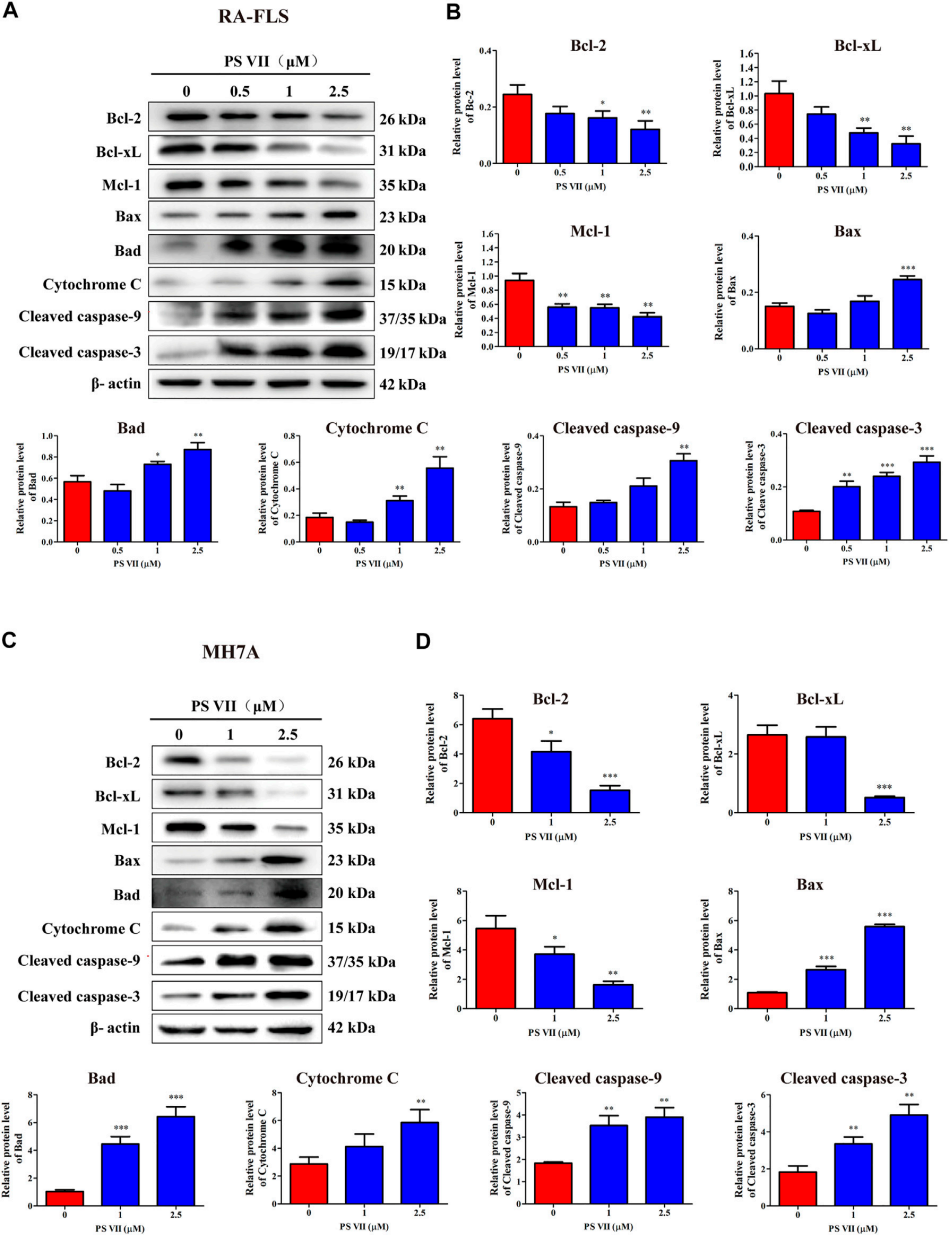
Effects of PS VII on the expression level of mitochondrial apoptosis pathway related proteins in RA-FLS and MH7A cells. Cells were treated with the indicated concentrations of PS VII for 48 h. The expression of apoptosis-related proteins in RA-FLS **(A)** and MH7A **(C)** was detected by western blot analysis. **(B, D)** Relative protein expression was determined by optical density analysis. Image J was used to determine the density ratio of each protein band relative to β-actin band. Compared with the control group, *p < 0.05, **p < 0.01, ***p < 0.001.

The mitochondrial apoptosis pathway plays a critical role in the apoptosis process of RA-FLS and is controlled by the Bcl-2 protein family (Estaquier et al., 2012). The effects of PS VII on the related proteins in the mitochondrial apoptosis pathway were detected by Western blot analysis. Figures 5A–D showed that PS VII treatment increased the expression levels of the pro-apoptotic proteins Bax and Bad, while the expression levels of the anti-apoptotic proteins Bcl-2, Bcl-xL, and Mcl-1 all decreased. Then, the process indicated that the integrity of mitochondria was impaired, which promoted the release of cytochrome C to cytoplasm, and induced the expression of Cleaved caspase-9 and Cleaved caspase-3. The results suggested that caspase-9 and caspase-3 were involved in PS VII mediated apoptosis. All together, our results proposed that the intrinsic mitochondrial pathway may be involved in PS VII induced apoptosis in RA-FLS and MH7A cells.

### PS VII Activated JNK and p38 Pathways in RA-FLS and MH7A Cells

**FIGURE 6:**
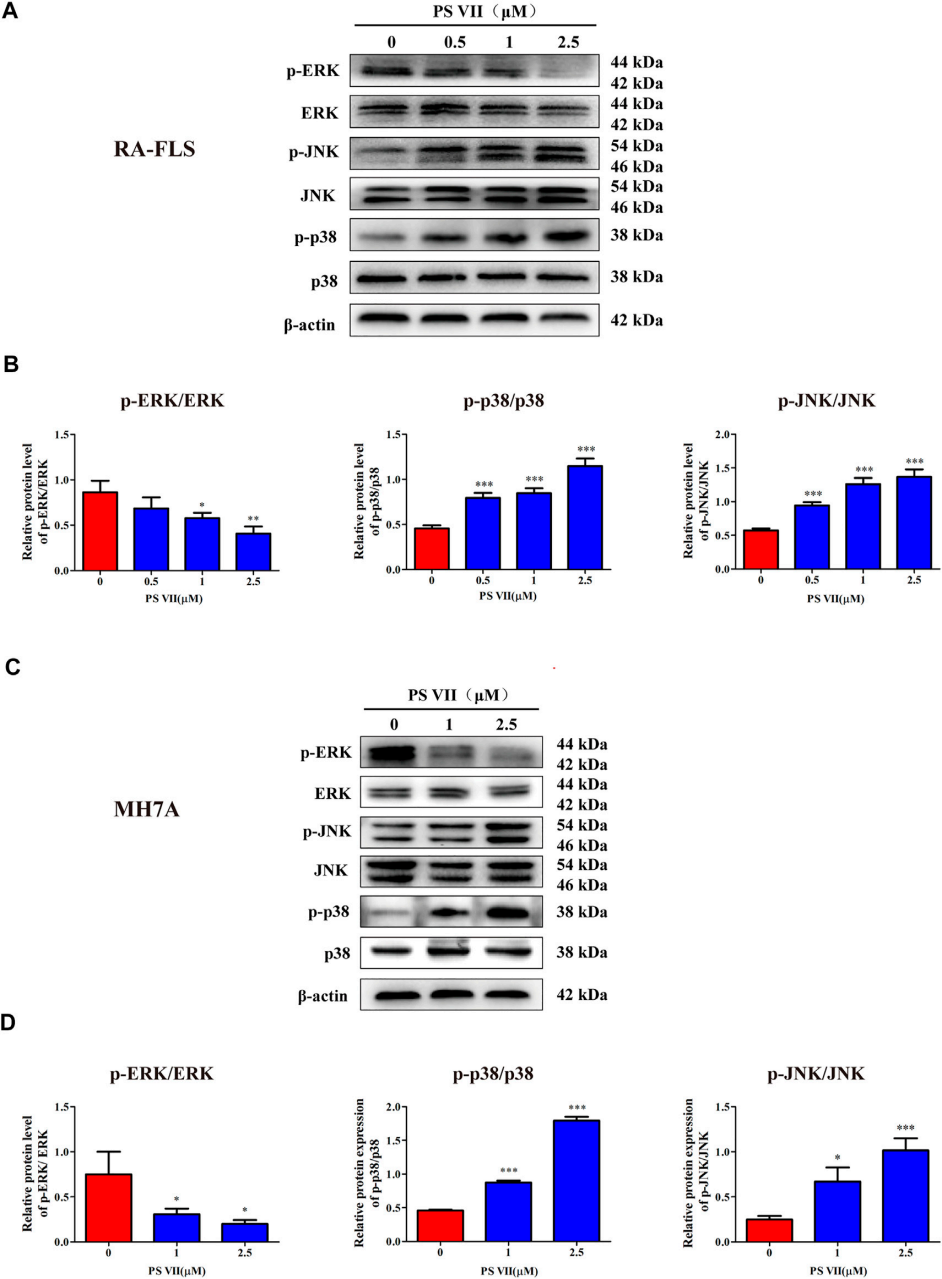
Effects of PS VII on the MAPK pathway in RA-FLS and MH7A cells. Cells were treated with the indicated concentrations of PS VII for 48 h. The expression of p-ERK, ERK, p-JNK, JNK, p-p38 and p38 protein in RA-FLS **(A)** and MH7A **(C)** was detected by western blot analysis. **(B, D)** The relative expression of proteins in RA-FLS and MH7A cells was determined by optical density analysis. Image J was used to determine the density ratio of each phosphorylated MAPK band relative to total protein content. Compared with the control group, *p < 0.05, **p < 0.01, ***p < 0.001.

The MAPK signaling pathway is involved in the regulation of a range of physiological processes, including apoptosis and proliferation, regulating this pathway is of great significance for the treatment of RA (Zhang et al., 2019). To elucidate whether the inhibitory effect of PS VII on proliferation and the induction of apoptosis was regulated by MAPK signaling pathway, we analyzed the expression of proteins related to the MAPK signaling pathway by western blot. As shown in Figures 6A–D, the expression of *p*-JNK and p-p38 proteins was increased, while the expression of *p*-ERK was reduced in RA-FLS and MH7A cells with PS VII treatment. The results suggested that PS VII may induce apoptosis in RA-FLS and MH7A cells by activating the JNK and p38 MPAK pathways.

**FIGURE 7:**
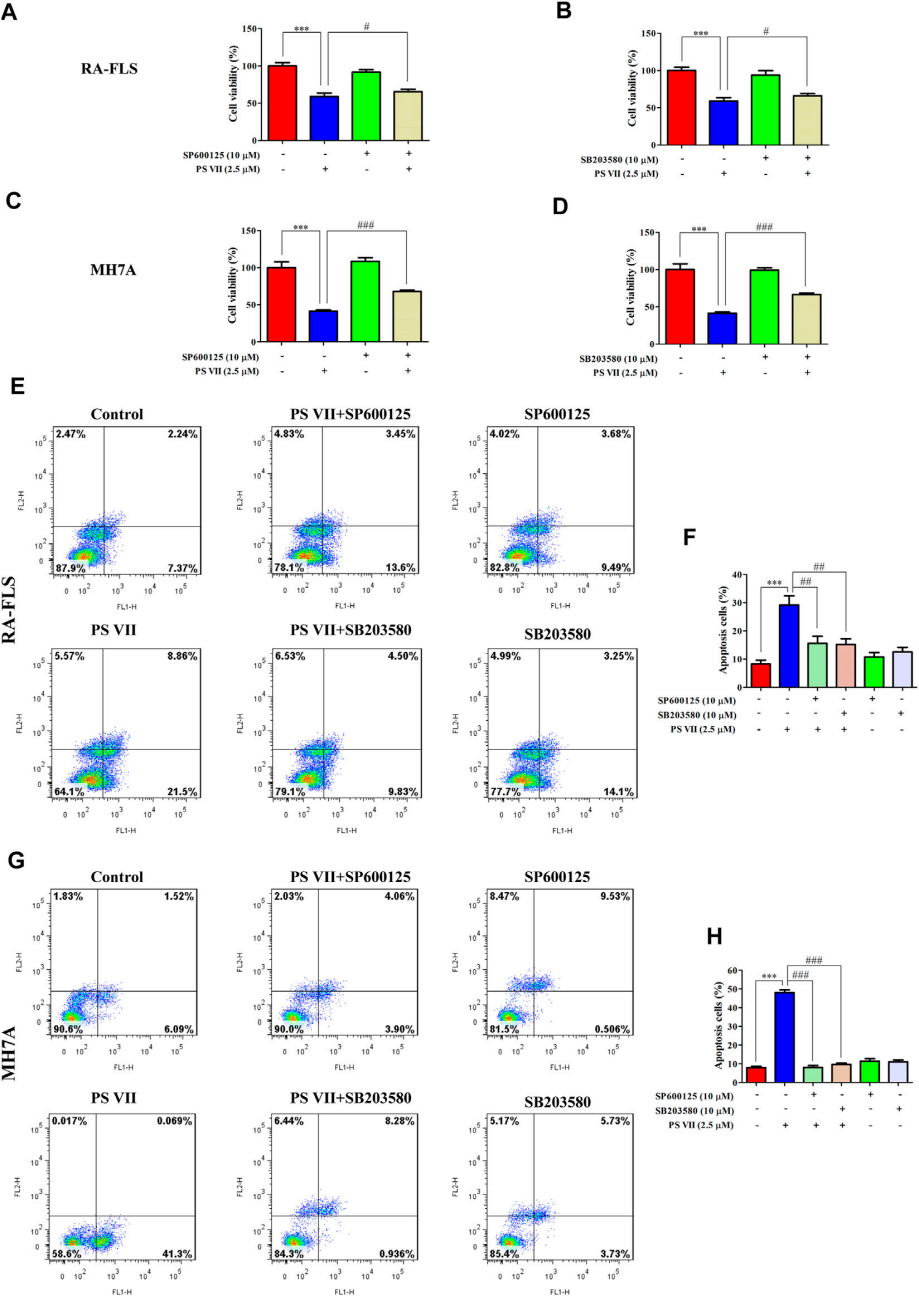
Effects of PS VII and inhibitors of JNK (SP6001256), p38 (SB203580) on the cell proliferation and apoptosis of RA-FLS and MH7A cells. Cells were treated with PS VII (2.5 μM), SP600125 (10 μM), SB203580 (10 μM) or SP600125, and PS VII, SB203580 and PS VII were co-cultured for 48 h. **(A, B)** Cell viability of RA-FLS cells was determined by MTT assay. **(C, D)** Cell viability of MH7A cells was determined by MTT assay. Flow cytometry was used to determine the extent of apoptosis in RA-FLS **(E)** and MH7A cells **(G)**. **(F, H)** The populations of apoptotic cells were evaluated as the percentage of total cells. Compared with the control group, ***p < 0.001. Compared with the PS VII group, ^#^ *p* < 0.05, ^##^ *p* < 0.01, ^###^ *p* < 0.001.

**FIGURE 8:**
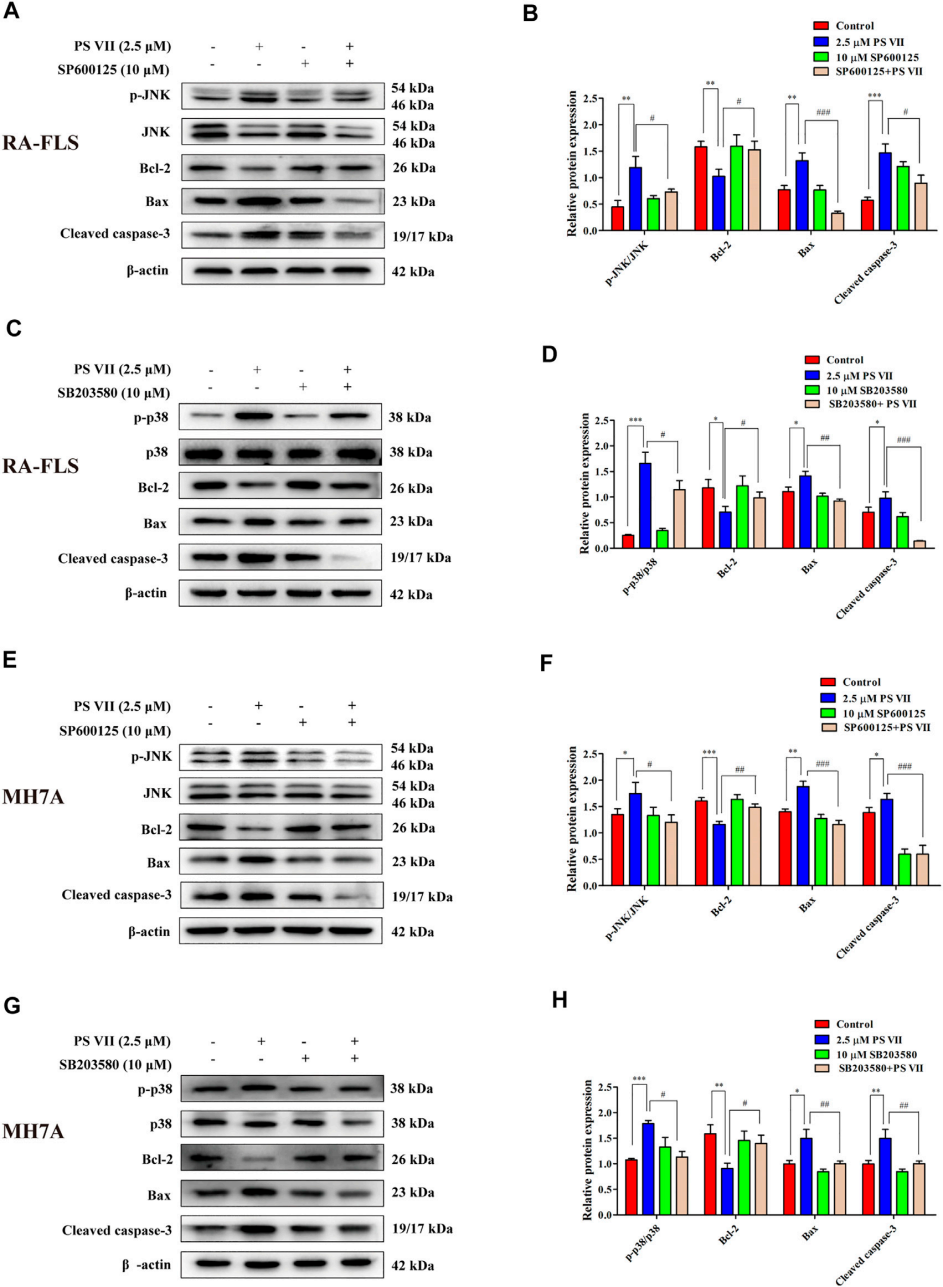
Effects of PS VII and inhibitors of JNK (SP6001256), p38 (SB203580) on apoptosis-related proteins of RA-FLS and MH7A cells. The expression of *p*-JNK, JNK, p-p38, p38, Bax, Bcl-2, and Cleaved caspase-3 in RA-FLS **(A, C)** and MH7A cells **(E, G)** was determined by western blot analysis. Relative protein expression in RA-FLS **(B, D)** and MH7A cells **(F, H)** was determined by optical density analysis. Image J was used to determine the density ratio of the *p*-JNK, p-p38 band relative to total protein content in RA-FLS and MH7A cells, and the density ratio of the Bcl-2, Bax, and Cleaved caspase-3 bands relative to β-actin bands. Compared with the control group, *p < 0.05, **p < 0.01, ***p < 0.001. Compared with the PS VII group, ^#^ *p* < 0.05, ^##^ *p* < 0.01, ^###^ *p* < 0.001.

JNK and p38 were the main factors involved in the MAPK signaling pathway, the activation of these proteins were essential for apoptosis (Yue and López, 2020). To further confirm whether the apoptosis induced by PS VII depends on the JNK and p38 MAPK signaling pathways, RA-FLS and MH7A cells were pretreated with JNK, p38 inhibitors (SP600125, SB203580). Then, treated with PS VII (2.5 μM). MTT analysis showed that the combination of PS VII with SP600125 or SB203580 could dramatically reverse the inhibitory effect of PS VII on the proliferation of RA-FLS and MH7A cells (Figures 7A–D). Also, flow cytometry result showed that the combination of PS VII with SP600125 or SB203580 partially reversed the levels of apoptosis induced by PS VII in both cells as compared to the effect of PS VII alone (Figures 7E–H). Consistent with these findings, western blot results showed that the combination of PS VII with SP600125 or SB203580 could result in the expression levels of the pro-apoptotic proteins Bax and Cleaved caspase-3 were reduced. In contrast, the expression of the anti-apoptosis protein Bcl-2 was increased in RA-FLS and MH7A cells (Figures 8A–H). Collectively, these results suggested that PS VII induced apoptosis were involved with the activation of JNK and p38 MAPK signaling pathways.

### PS VII Ameliorated Body Weight, Paw Swelling, Ankle Joint Diameter, and Arthritis Index of AIA Rats

**FIGURE 9:**
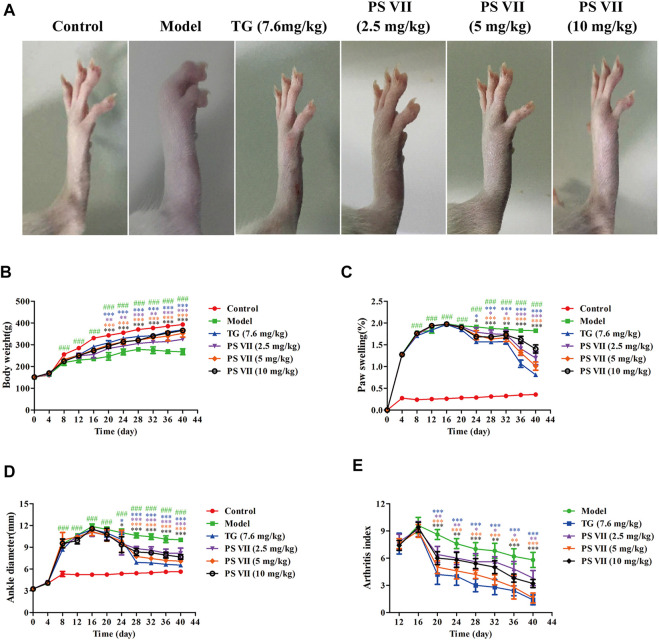
Effects of PS VII on the severity of AIA rats. After the establishment of the AIA model, rats were intragastrically administered PS VII (2.5 mg/kg, 5 mg/kg, 10 mg/kg) and TG (7.6 mg/kg) every day. The control group and the model group were given an equal volume of 0.5% CMC-Na. **(A)** Morphological representation of the right hind paw of rats from six groups on the day 40 after CFA injection. Observe body weight **(B)**, paw swelling **(C)**, ankle joint diameter **(D)**, and arthritis index **(E)** every 4 days. Data were expressed as mean ± SD (*n* = 5). Compared with the control group, ^###^ *p* < 0.5, compared with the model group, *p < 0.05, **p < 0.01, ***p < 0.001.

As shown in Figure 9A, the rat’s right hind paw was obviously swollen in the model group. TG and PS VII significantly inhibited the swelling of the right hind paw of rats. The weight of rats in the control group gradually increased during the entire experiment. From the day 8, the weight gain of the model group was significantly lower than that of the control group. Compared with the model group, the treatment of PS VII (2.5 mg/kg, 5 mg/kg, 10 mg/kg) could partially reduce the degree of weight loss, and the body weight increased gradually from the day 12 (Figure 9B).

PS VII (2.5 mg/kg, 5 mg/kg, 10 mg/kg) significantly reduced paw swelling and ankle joint diameter in AIA rats during the entire drug treatment (Figures 9C,D). After CFA immunization, the inflammatory symptoms gradually aggravated with the swelling of the toes, and the arthritis index reached its peak on the day 16. Similarly, the arthritis index decreased significantly after PS VII treatment (Figure 9E).

### PS VII Reduced the Spleen and Thymus Index of AIA Rats

**FIGURE 10:**
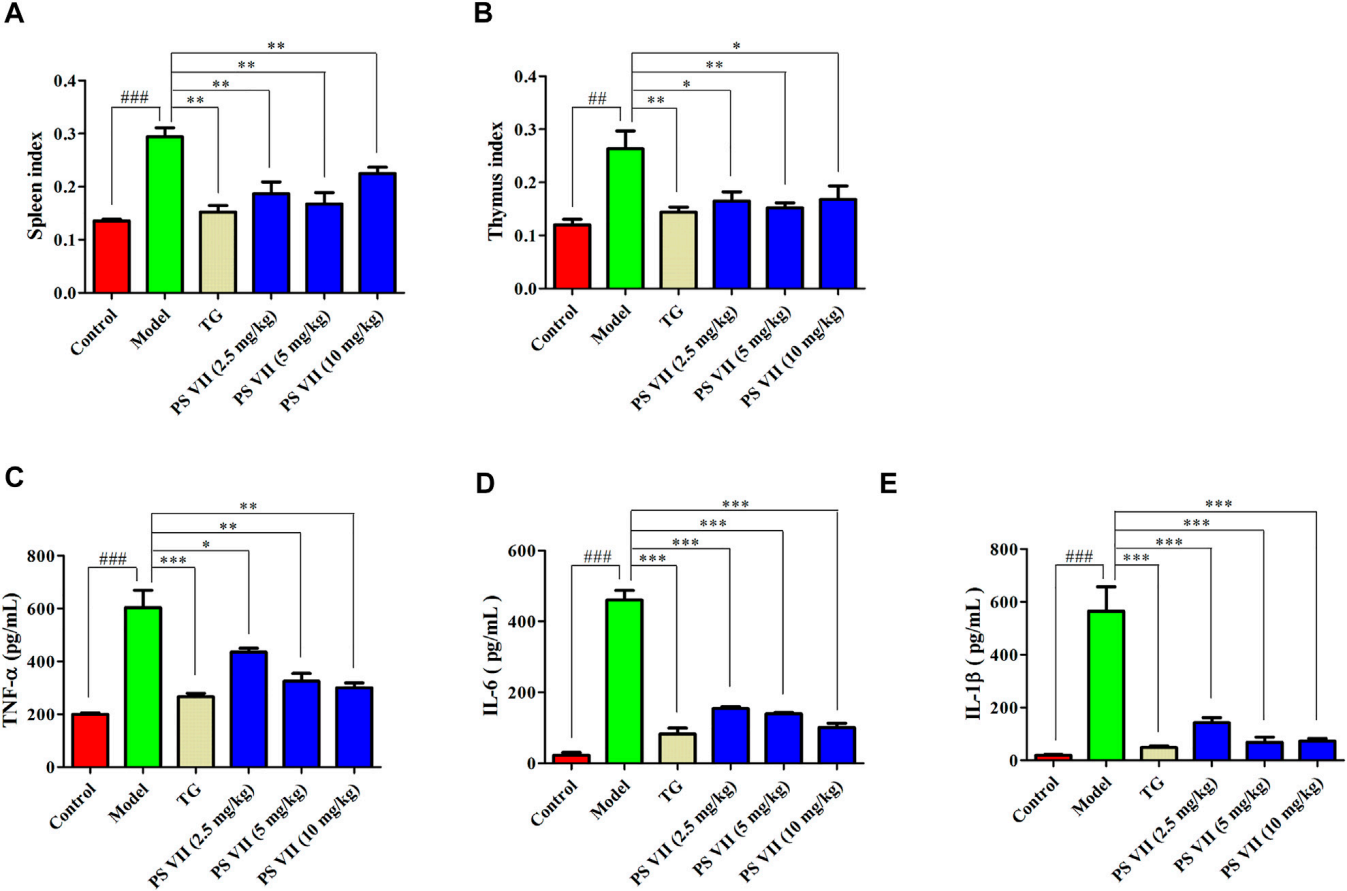
Effects of PS VII on the spleen, thymus index and serum levels of inflammatory cytokines. **(A)** Spleen index. **(B)** Thymus index. The production of TNF-α **(C)**, IL-6 **(D)**, and IL-1β **(E)** in the serum of AIA rats. Data were expressed as mean ± SD, *n* = 5. Compared with the control group, ^#^ *p* < 0.05, ^*##*^ *p* < 0.01, compared with the model group, *p < 0.05, **p < 0.01, ***p < 0.001.

The spleen and thymus of rats were weighed to calculate the rat’s spleen and thymus index (Figure 10A,B). There were significant differences in spleen and thymus indexes between the model group and the control group. Compared with the model group, TG and PS VII treatment could significantly reduce the thymus and spleen indexes of AIA rats.

### PS VII Suppressed the Production Pro-inflammatory Cytokines in AIA Rats

As shown in Figures 10C–E, in the model group, the production of pro-inflammatory cytokines including TNF-α, IL-6, and IL-1β were increased compared with the control group. Nevertheless, both TG and PS VII treatment could decrease those pro-inflammatory cytokines levels.

### PS VII Improved Histopathological Changes in AIA Rats

**FIGURE 11:**
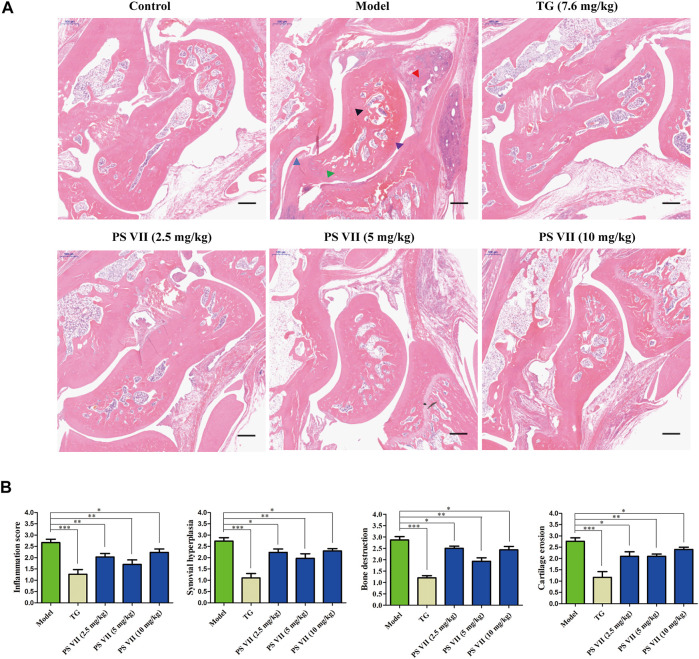
Effects of PS VII on the pathological changes of AIA rats. **(A)** Hematoxylin and eosin (HE) stained representative sections of rat ankle joints from different groups. **(B)** Determine the histological score as described in the Materials and Methods section. Purple arrow: joint cavity; blue arrow: synovial hyperplasia; red arrow: inflammatory cell infiltration; black arrow: bone erosion; green arrow: cartilage destruction. Data are expressed as mean ± SD (*n* = 3). Compared with the model group, *p < 0.05, **p < 0.01 and ***p < 0.001.

To further assess the effect of PS VII on developed arthritis, a histopathological evaluation was performed. As shown in Figure 11A, no pathological changes of arthritis were observed in normal joints. In contrast, the model group slices clearly observed the pathological features of RA, including inflammatory cell infiltration, synovial hyperplasia, bone, and cartilage erosion. However, TG and PS VII treatment could improve the above-mentioned pathological changes (Figures 11A,B). These results further demonstrated the anti-RA effect of PS VII.

### PS VII Regulated the Proteins Expression in Synovial Tissues

**FIGURE 12:**
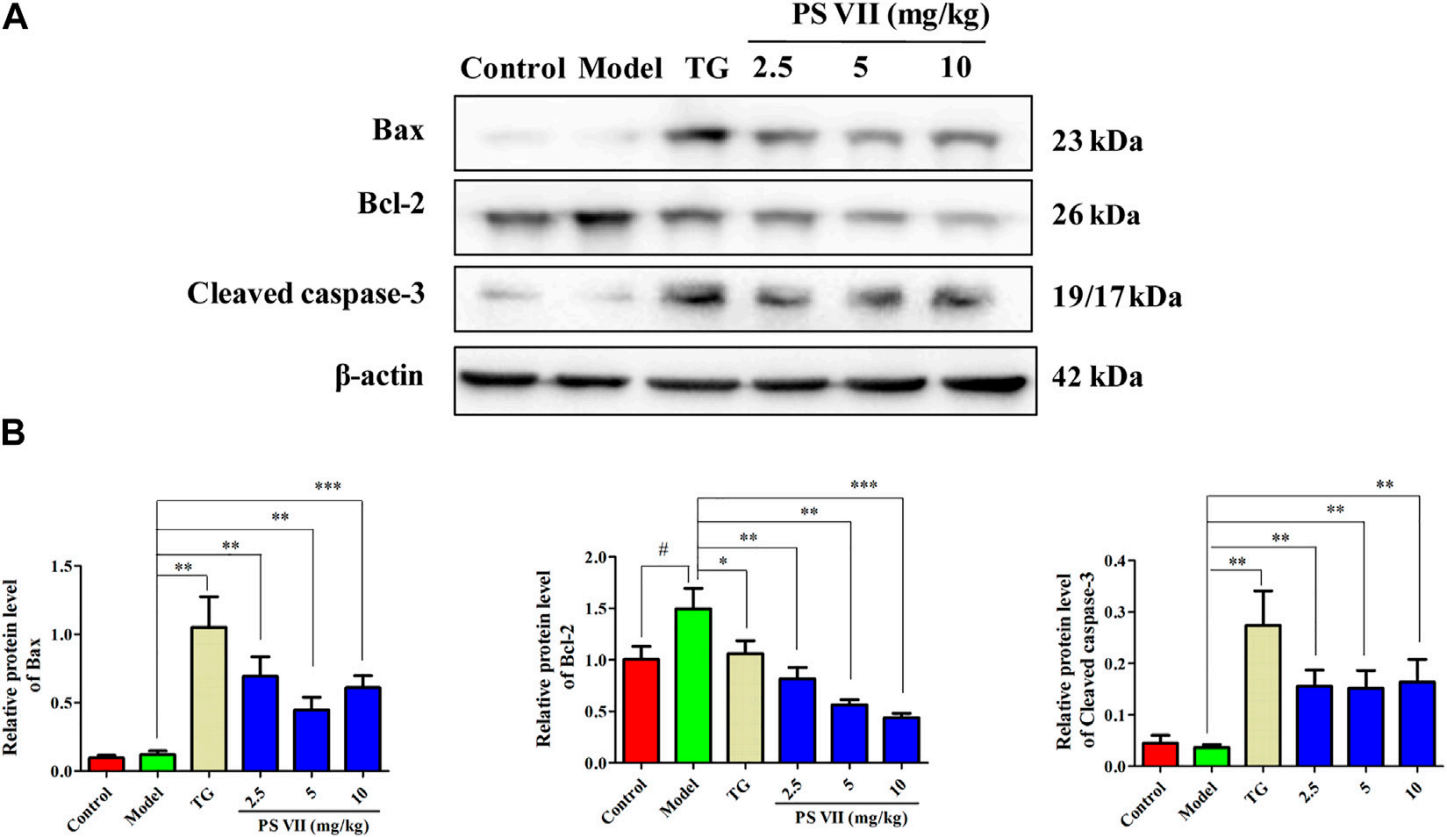
Effects of PS VII on proteins in the synovial tissues of experimental rats. **(A)** The expression of Bax, Bcl-2, Cleaved caspase-3 in synovial tissues was determined by western blot analysis. **(B)** Relative protein expression was determined by optical density analysis. Image J was used to determine the density ratio of each protein band relative to β-actin band. Data were expressed as mean ± SD, *n* = 5. Compared with the control group, ^#^ *p* < 0.05, ^*##*^ *p* < 0.01, compared with the model group, *p < 0.05, **p < 0.01, ***p < 0.001.

The expression levels of Bax, Bcl-2, and Cleaved caspase-3 in the synovial tissues of experimental rats were detected (Figures 12A,B). Results showed the increased expression of Bax, Cleaved caspase-3, and the decreased expression of Bcl-2 in the synovial tissues with PS VII treatment. These results were further strengthened the therapeutic effect of PS VII in addition to *in vitro* findings.

## Discussion

RA is a chronic autoimmune disease with synovitis and synovial hyperplasia as the main pathological features (Josef et al., 2018). The pathological process of RA involves a variety of cells, including B cells, T cells, macrophages, and FLS (McInnes and Schett, 2017; Wu et al., 2021). FLS is a highly specific mesenchymal cell that exists in the synovium of joints. In healthy individuals, normal FLS as the producer of the components for synovial fluid and intracellular matrix, the modulator for synovial fluid content, the promotor of joint repair, the controller for joint inflammation, maintains the structural integrity of the synovial lining and normal joint balance (Bartok and Firesteinet, 2010). Increasing evidence demonstrated that activated RA-FLS plays a vital role in the pathogenesis progression of RA. At the pathological conditions, the abnormal proliferation of RA-FLS interferes with the balance between cell survival and death (Bottini and Firesteinet, 2013). Moreover, RA-FLS usually appears resistance to apoptosis in RA patients (Pope, 2002). In addition to promoting the inflammatory environment, RA-FLS also produce matrix metalloproteinases, which degrade extracellular matrix and promote cartilage destruction. Based on the crucial role of RA-FLS in the occurrence of synovial hyperplasia and destructive arthritis, new drugs targeting to RA-FLS is considered to be new therapeutic strategies for RA therapy (Zhang et al., 2019).

Steroidal saponins distributed in various herbal medicines, was clarified to present excellent pharmacological activity, especially the cytotoxic activity on multiple cancer cells. PS VII as one of the steroidal saponins, is extracted from *Trillium tschonoskii* Maxim. As confirmed in the literature, PS VII could induce apoptosis and cell cycle arrest by regulating Ras pathway, thereby inhibiting the growth of colorectal cancer cells (Li et al., 2014b). Also, PS VII could inhibit the proliferation of Hela cells and promote apoptosis through endogenous apoptosis pathway (Zhang et al., 2014). Another study has shown that PS VII treatment induces apoptosis by promoting mitochondrial-mediated ROS production and activating MAPK pathway, and inhibits the growth of HepG2 cells (Zhang et al., 2016). Besides, PS VII was found to induce caspase dependent apoptosis and autophagy through AMPK-ULK1 pathway, thus inhibiting the growth of NSCLC cells (Qian et al., 2020). So, we tested the inhibitory activity of PS VII on RA-FLS and MH7A cells due to the similar characteristics of RA-FLS as tumor cells. As expected, PS VII significantly inhibited the proliferation of RA-FLS and MH7A cells.

Cell proliferation is regulated by cell cycle progression, while apoptosis is related to cell cycle arrest (Vermeulen et al., 2003). It is known to all that the expression of the cyclin/cyclin-dependent kinase complex plays a pivotal role in S phase progression. The binding of cyclin A to CDK2 is necessary for cells enter into the S phase. The CDK inhibitor p21 intercepts the interaction of the cyclin A2 and CDK2, leading to cell cycle arrest in S phase (Karimian et al., 2016). Previous studies have demonstrated that PS VII induced G0/G1 phase arrest in colorectal cells or G2/M phase arrest in lung adenocarcinoma cells. The mechanism was revealed to inhibit CDK4/CDK6-cyclinD1 complex by increasing p21 expression in colorectal cells, and upregulate p21 expression level in lung adenocarcinoma cells (Li et al., 2014b; Zhao et al., 2016). Our results showed that PS VII treatment caused RA-FLS and MH7A cells to arrest in S phase, and the protein levels of cyclin A2 and CDK2 were downregulated. In contrast, the protein levels of P21 and P27 were upregulated. These results demonstrated that PS VII treatment inhibited the expression of cyclin A2 and related CDK2 activities, and ultimately induced S phase cell cycle arrest.

Apoptosis resistance is the main reason for the abnormal proliferation of RA-FLS. Existing evidence proposed that the induction of apoptosis in RA-FLS would be beneficial for controlling the symptoms and development of RA patients (Zhang et al., 2019). In the present study, we found that the morphological changes took place in cell nuclei and apoptosis occurred after PS VII treatment. Apoptosis is regulated predominantly through two signal transduction pathways: the intrinsic pathway depended on the mitochondrial apoptosis cascade, or the extrinsic pathway related to the mediation of death receptors (Obeng, 2021). The family of Bcl-2 proteins plays a pivotal role in modulating apoptosis in RA-FLS cells. Bcl-2 proteins regulated apoptosis via mitochondrial-related apoptosis pathways, in which members of the anti-apoptotic family of Bcl-2 proteins (such as Bcl-xL and Bcl-2) inhibited apoptosis, while pro-apoptotic proteins (such as Bax and Bad) activated apoptosis (Singh et al., 2019). Reducing the ratio of anti-apoptotic factors/pro-apoptotic factors (Bcl-2/Bax) may lead to the release of mitochondrial cytochromes and the activation of caspase-9, caspase-3; and then cleave intracellular substrates, including PARP, ultimately induce apoptosis (Bock et al., 2020). Our results indicated that PS VII treatment could downregulate the expression of Bcl-2, Bcl-xL, and Mcl-1 proteins, upregulate the expression of Bax and Bad proteins. We also found that the production of cytochrome C in the cytoplasm was increased, along with the raised levels of both cleaved caspase-9 and cleaved caspase-3. Collectively, the molecular mechanism underlying PS VII induced apoptosis in RA-FLS and MH7A cells were involved in the mitochondria-mediated intrinsic pathway.

The MAPK signaling pathway could regulate cell proliferation, apoptosis, cytokine expression, and matrix metalloproteinases in RA-FLS (Zhang et al., 2019). MAPK family members expressed in synovial tissue and FLS, mainly includes ERK, p38, and JNK. The ERK pathway promotes the production of TNF-α and IL-1 by stimulating the proliferation of T cells to recruit and activate monocytes and macrophages. Then, a variety of inflammatory factors are released from synovial tissue, thereby, promoting the abnormal proliferation of RA-FLS. JNK activation acts as a double-edged sword in cell apoptosis. The effect of JNK activation on cell apoptosis depends on cell type, stimulation method and the activity of other signal transduction pathways (Neff et al., 2003). Activation of the p38 MAPK pathway usually contributes to cell apoptosis (Yue and López et, 2020). The cytotoxicity of PS VII on cancer cells was closely related to the MAPK signaling pathway. For example, PS VII inhibited the migration and invasion of osteosarcoma cells by inhibiting the p38 MAPK pathway to produce MMP-2, 9 (Cheng et al., 2016). In addition, PS VII induced HepG2 cell apoptosis through modulating ROS-mediated mitochondrial dysfunction and MAPK pathway (Zhang et al., 2016). Several natural products induced RA-FLS apoptosis by activating JNK and p38 phosphorylation and inhibiting ERK phosphorylation, while blocking p38 and JNK signaling partially reversed this effect (Liagre et al., 2007; Zhang et al., 2019). Besides, the apoptosis induced by RA-FLS was mediated by activation of p38 without affecting JNK and ERK pathways (Zou et al., 2016). In this work, the results revealed that PS VII treatment increased the phosphorylation of JNK and p38, decreased phosphorylation of ERK. Notably, the addition of the JNK inhibitor (SP600125), and the p38 inhibitor (SB203580), obviously reversed the inhibitory effect on cell proliferation and the induction of apoptotic with PS VII treatment. These results suggested that PS VII induced apoptosis mainly through regulating the JNK and P38 MAPK signaling pathway in RA-FLS and MH7A cells.

**FIGURE 13:**
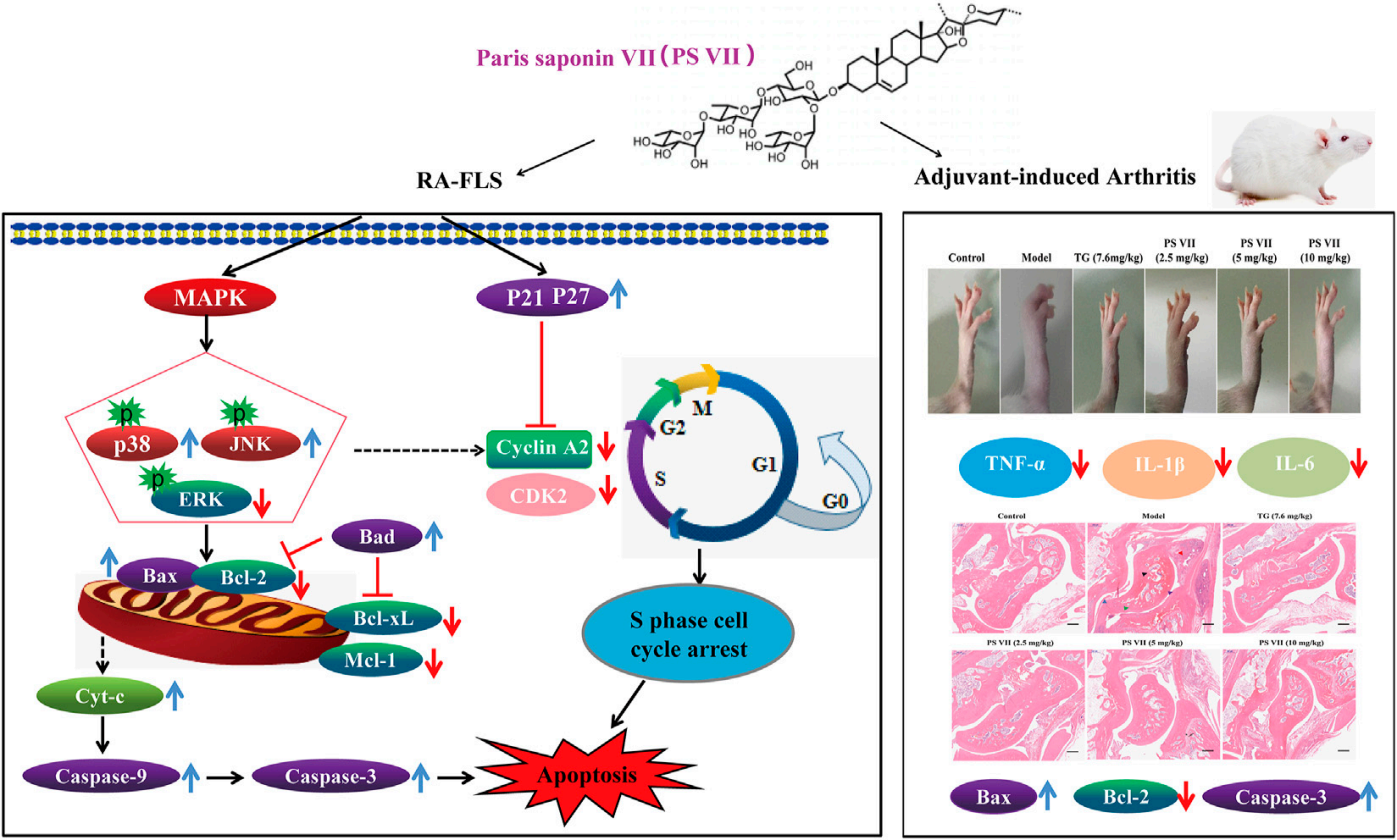
Schematic representation of proposed mechanism responsible for PS VI-mediated treatment of rheumatoid arthritis.

It is well known that the AIA rats has some similar pathological symptoms to RA, such as inflammatory cell infiltration, synovial hyperplasia, increased paw, and foot volume, cartilage destruction and bone erosion (Choudhary et al., 2018). Studies have shown that the weight, toe swelling degree and AI score of rats are important indicators for evaluating inflammation (Wang et al., 2016). In this work, it was found that the body weight of PS VII-treated AIA rats continued to increase and eventually approached the normal level, improved the paw swelling of AIA rats, reduced the ankle joint diameter and AI score, indicating that PS VII could alleviate the main symptoms of RA. In addition, PS VII could reduce the thymus and spleen index. Pro-inflammatory cytokines in RA are the main cause of inflammation and joint destruction, such as TNF-α, IL-6, and IL-1β (Mateen et al., 2016). The levels of TNF-α, IL-6, and IL-1β in serum were evaluated to assess the severity of RA. This work demonstrated that the levels of TNF-α, IL-6, and IL-1β in the serum of AIA rats were significantly increased, indicating that AIA rats caused severe inflammation. As expected, PS VII treatment suppressed the secretion of these inflammatory cytokines. Histopathology provides prominent morphological and pathological features of RA (Zhai et al., 2019; Wang et al., 2020). Rats in the model group were observed for inflammatory cell infiltration, synovial tissue hyperplasia, articular cartilage and bone erosion. The treatment of PS VII could improve histopathological changes, which further demonstrated the protection effects of PS VII on RA. Moreover, the treatment of PS VII could also upregulate the expression of apoptosis proteins Bax, Cleaved caspase-3, and down-regulate Bcl-2 in the synovial tissues of experimental rats. The results were further strengthened the therapeutic effect of PS VII in addition to *in vitro* findings. The study has demonstrated the anti-rheumatoid arthritic effects of PS VII *in vitro* and *in vivo* (Figure 13).

## Conclusion

In summary, we demonstrated that the anti-RA effects of PS VII in human rheumatoid arthritis fibroblast-like synoviocytes and AIA in rats. *In vitro*, PS VII could inhibit cell proliferation, induce cell cycle arrest and apoptosis by regulating the mitochondria-mediated intrinsic pathway and activating the JNK and p38 MAPK pathways. *In vivo*, PS VII could ameliorate body weight, paw swelling, ankle joint diameter, reduce the spleen and thymus index, suppress the production pro-inflammatory cytokines, improve histopathological changes and regulate expressions of apoptosis proteins in AIA Rats. These findings indicated that PS VII has great potential for drug development in the treatment of RA.

## Data Availability

The original contributions presented in the study are included in the article/Supplementary Material, further inquiries can be directed to the corresponding authors.
